# Identification and Expression Analysis of NAC Transcription Factors Related to Rust Resistance in Foxtail Millet

**DOI:** 10.3390/plants14101507

**Published:** 2025-05-17

**Authors:** Keke Gong, Jia Liu, Mengya Zhang, Zhiping Dong, Jifang Ma, Peixue Xuan, Hui Bai, Zhiyong Li

**Affiliations:** 1 Institute of Millet Crops, Hebei Academy of Agriculture and Forestry Sciences, Shijiazhuang 050035, China; g1752993549@163.com (K.G.);; 2 Key Laboratory of Genetic Improvement and Utilization for Featured Coarse Cereals (Co-Construction by Ministry and Province), Ministry of Agriculture and Rural Affairs, Institute of Millet Crops, Hebei Academy of Agriculture and Forestry Sciences, Shijiazhuang 050035, China; 3 Key Laboratory of Minor Cereal Crops of Hebei Province, Institute of Millet Crops, Hebei Academy of Agriculture and Forestry Sciences, Shijiazhuang 050035, China

**Keywords:** *Setaria italica*, NAC, *Uromyces setariae-italicae*, transcription factor, rust disease resistance, qRT-PCR

## Abstract

Foxtail millet (*Setaria italica*), a vital cereal crop in China, serves as both a staple food and forage source but is threatened by rust disease caused by *Uromyces setariae-italicae* (*Usi*), leading to severe yield and quality losses. The NAM, ATAF1/2, and CUC2 (NAC) transcription factor family represents one of the largest plant-specific regulatory gene families, playing pivotal roles in development and stress responses. However, the functional relevance of NAC genes in foxtail millet’s defense against this pathogen remains unexplored. Here, we systematically analyzed 33 *SiNAC* genes from the foxtail millet genome. Phylogenetic analysis classified these genes into 11 subgroups, while chromosomal mapping localized them to nine chromosomes unevenly. Promoter analysis identified stress- and plant hormone-related *cis*-elements, suggesting functional diversity. Expression profiling analysis showed that most *SiNAC* genes exhibit tissue-specific expression patterns. Quantitative real-time PCR (qRT-PCR) results indicated that 30 genes responded to *Usi* infection, with 17 showing a strong association with rust resistance. Three resistance-associated genes demonstrated transactivation activity and nuclear localization, indicating their regulatory function in defense responses. This study provides both mechanistic insights into *SiNAC*-mediated rust resistance and potential targets for molecular breeding in foxtail millet.

## 1. Introduction

Foxtail millet, originating in China, exhibits remarkable characteristics, such as drought resistance, tolerance to barrenness, and rich nutrition. It is one of the most important cereal crops in China. However, rust disease, caused by *Usi*, is one of the most common diseases in foxtail millet and can trigger epidemics, resulting in yield losses of 10–30% [1,2]. Although transcription factors (TFs) have been extensively documented to play pivotal roles in plant–pathogen interactions, their involvement in the foxtail millet-*Usi* pathosystem remains unexplored to date.

NAC (NAM, ATAF1/2, and CUC2), one of the largest plant-specific transcription factor families, plays an important role in plant growth and development, morphogenesis, hormone signal regulation, and responses to biotic and abiotic stresses [3,4,5]. Souer et al. [6] cloned the first NAC transcription factor from *Petunia* in 1996. Since then, with the completion of genome sequencing, further NAC transcription factors have been identified in plants, such as *Arabidopsis* [7], barley [8], wheat [9], rice [10], maize [11], oat [12], cotton [13], and foxtail millet [14].

Numerous studies have highlighted the roles of NAC transcription factors in plant abiotic stress responses. Overexpression of *OsNAC2* increases plant sensitivity to NaCl stress, leading to salt-induced cell death marked by plasma membrane disruption, nuclear DNA fragmentation, and altered caspase-like activity [15]. In wheat, *TaNAC071-A* overexpression significantly boosts drought tolerance by enhancing water-use efficiency and upregulating stress-responsive genes, indicating its positive regulation of drought response [16]. Under drought conditions, *GmNAC12*-overexpressing soybean lines show a survival rate increase of over 57% compared to wild types, suggesting *GmNAC12*’s positive role in drought stress regulation [17].

In addition to abiotic stresses, NAC genes also play critical roles in biotic stresses. *OsNAC58* overexpression in rice increases resistance to bacterial leaf blight [18]. In wheat, *TaNAC6* may participate in basal and broad-spectrum resistance to wheat powdery mildew through the JA signaling pathway [19]. *Rph7* enhances resistance by activating basal defense mechanisms, modulating the expression of downstream genes, and inducing partial cell death [20]. *SlNAP1* directly turns on other genes that help deactivate gibberellin (GA) and produce salicylic acid (SA) and abscisic acid (ABA). This boosts the tomatoes’ defenses against leaf speck disease and bacterial wilt disease [21]. Virus-induced gene silencing (VIGS) analysis in tomatoes revealed that *SlNAC61* plays a positive role in the plant’s response to infection stress caused by Tomato Yellow Leaf Curl Virus (TYLCV) [22]. Transgenic rice plants with overexpressing *OsNAC111* show high expression levels of defense-related genes, resulting in increased resistance to rice blast fungus [23].

Numerous studies highlight that NAC genes can play dual roles in both biotic and abiotic stresses. For example, overexpression of *GhATAF1* in cotton plant enhances tolerance to salt stress, but attenuates the resistance to the fungal pathogens *V. dahliae* and *Botrytis cinerea* [24]. In pepper, *CaNAC4* overexpression enhances the plant’s tolerance to salt stress, but simultaneously increases the susceptibility to infection by *Botrytis cinerea*, leading to more severe disease symptoms [25]. Grape *NAC61* improves postharvest dehydration tolerance but also increases disease severity from Botrytis cinerea infections, suggesting a dual role in osmotic, oxidative, and biotic stress responses [26]. In mungbean, the high expression of *VrNAC7*.7 is induced under bacterial leaf spot disease, powdery mildew, and drought stress conditions [27]. Tobacco *SlNAC35* upregulation under drought, salt, and bacterial pathogen stresses promotes root growth and also enhances bacterial resistance [28].

Mining disease-resistant gene resources and breeding rust-resistant foxtail millet varieties are the most cost-economical, efficient, and environmentally sustainable friendly strategies for controlling foxtail millet rust. China has a diverse collection of rust-resistant germplasm, but the genetic basis of resistance remains poorly understood in most of these accessions. In this study, we focus on the highly resistant cultivar Shilixiang (SLX), which carries a single major resistance gene (unpublished data). To elucidate the defense mechanisms mediated by NAC transcription factors in foxtail millet under rust disease (*Usi*) stress, we focused on 33 *SiNAC* genes previously identified as abiotic stress-responsive candidates [14]. First, we performed comprehensive bioinformatic analyses to characterize their phylogenetic relationships, chromosomal distributions, and *cis*-regulatory elements. Next, we systematically investigated their expression patterns through (1) developmental expression profiling across diverse tissues and growth stages, and (2) time-course qRT-PCR analysis in both resistant and susceptible cultivars following *Usi* inoculation. Finally, we functionally characterized three resistance-associated *SiNAC* genes (*SiNAC063*, *SiNAC070*, and *SiNAC118*) through nuclear localization assays and transcriptional activation tests, confirming their roles as functional transcription factors. This integrated approach provides novel insights into the potential roles of *SiNAC* genes in foxtail millet’s rust defense responses.

## 2. Results

### 2.1. Selection and Phylogeny Analysis of the SiNAC Genes

Puranik et al. [14] first reported that 50 *SiNAC* genes in foxtail millet showed differential expression patterns under various abiotic stress conditions, including dehydration, salinity, cold, and treatments with ABA, SA, methyl jasmonate (MeJA) and ethylene (Et). Building upon these findings, we selected 33 *SiNAC* genes for further characterization (Table S1).

**Figure 1:**
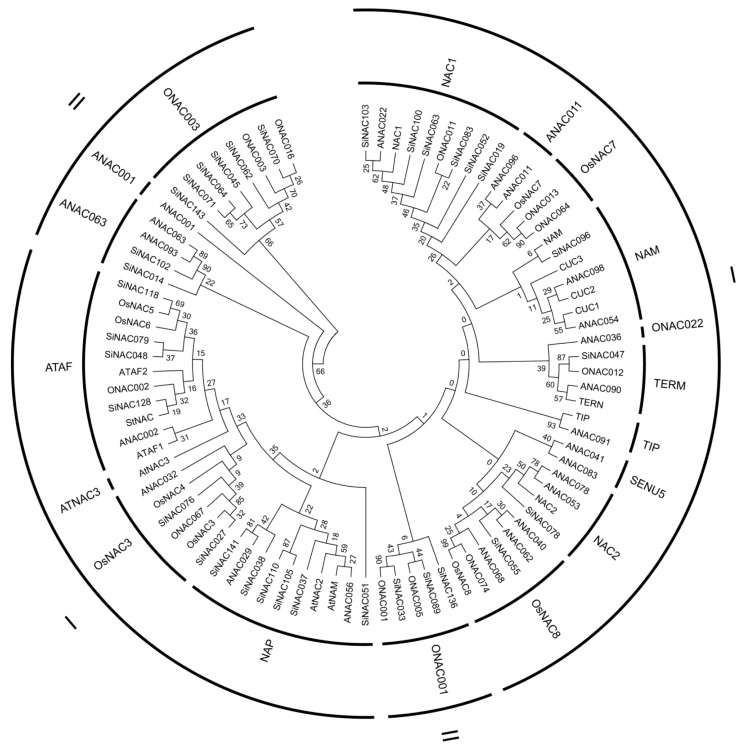
Phylogenetic tree of NAC genes in *Setaria italica*, *Oryza sativa*, and *Arabidopsis thaliana*.

Following the classification system established by Ooka et al. [7], the NAC family genes were systematically categorized into two major groups: Group I (containing 14 subgroups) and Group II (comprising 4 subgroups). To elucidate evolutionary relationships, we performed phylogenetic analysis using predicted NAC domains from the 33 selected *SiNACs* in *Setaria italica*, along with ONACs from *Oryza sativa*, ANACs from *Arabidopsis thaliana*, and other known NAC family proteins (Table S2, Figure 1). The phylogenetic reconstruction demonstrated that the 33 *SiNAC* genes were distributed across 11 distinct subgroups, while 7 subgroups (ANAC001, AtNAC3, SENU5, TIP, OsNAC7, ONAC022, and ANAC011) were not represented in our dataset. Notably, six SiNAC proteins clustered into three well-characterized subgroups: NAC1, ONAC003, and NAP. The ATAF subgroup contained four SiNAC proteins, while ONAC001, ANAC063, and OsNAC3 subgroups comprised three, two, and two SiNAC proteins, respectively. In contrast, the OsNAC8, NAC2, TERN, and NAM subgroups each contained only a single SiNAC protein.

### 2.2. Chromosomal Distribution of SiNAC Genes

**Figure 2:**
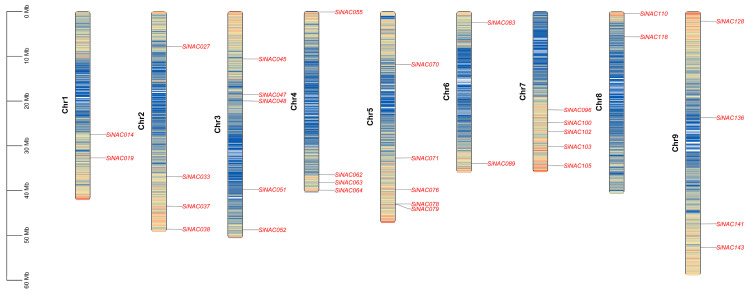
Chromosomal distribution of 33 *SiNAC* genes in *Setaria italica*. The left-hand scale denotes the genomic length in megabases (Mb).

Chromosomal localization analysis demonstrated an uneven distribution of the 33 *SiNAC* genes across nine foxtail millet chromosomes. Notably, chromosomes 3, 5, and 7 exhibited the highest gene density, each harboring five *SiNAC* genes. A moderate distribution was observed on chromosomes 2, 4, and 9, with four genes per chromosome. In comparison, chromosomes 1, 6, and 8 displayed the lowest *SiNAC* representation, containing only two genes each (Figure 2).

### 2.3. Cis-Regulatory Element Analysis of SiNAC Gene Promoter

**Figure 3:**
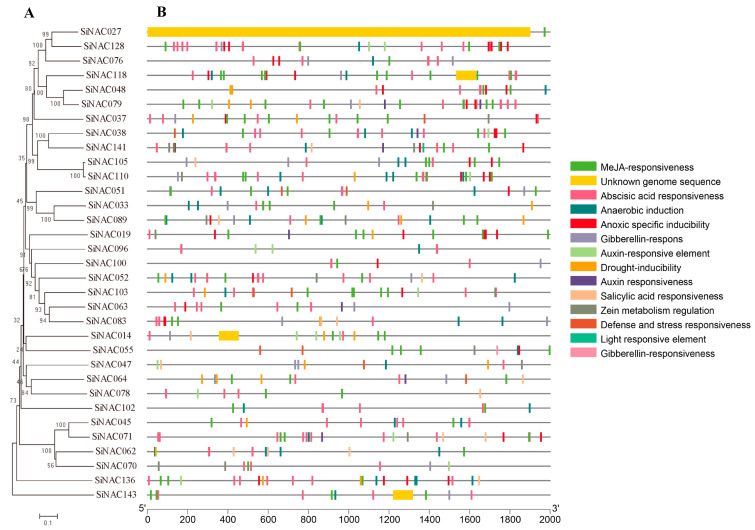
Prediction of *cis*-acting elements in the promoters of 33 *SiNAC* genes. (**A**) Phylogenetic tree of SiNACs. The phylogenetic relationships among homologous proteins were reconstructed using MEGA5 software, employing the neighbor-joining algorithm with bootstrap validation (n = 1000 replicates) to assess topological robustness. (**B**) *Cis*-acting element structures in promoter regions of *SiNACs*. Different colors represent 13 kinds of 196 functional modules and unknown genome sequence.

Transcriptional regulation of *SiNAC* genes is mediated by *cis*-acting elements located in their promoter regions. Comprehensive promoter analysis revealed 13 distinct *cis*-regulatory elements associated with stress responses, hormone signaling pathways, and developmental processes, in addition to the conserved CAAT-box and TATA-box core promoter elements (Figure 3).

32 of 33 *SiNAC* genes each contained 3–9 such elements except *SiNAC027*. A striking 94% (31/33) of the *SiNAC* promoters contained MeJA-responsive elements, with only *SiNAC047* and *SiNAC096* lacking these motifs. Notably, *SiNAC064* and *SiNAC141* promoters were enriched in defense- and stress-related *cis*-elements, whereas *SiNAC052* and *SiNAC062* contained multiple MYB-binding sites known to mediate drought-responsive gene expression. Additionally, five genes (*SiNAC052*, *SiNAC055*, *SiNAC062*, *SiNAC071*, and *SiNAC118*) possessed elements associated with anaerobic induction, four of which contained anaerobic-specific response motifs. All 33 *SiNAC* promoters contained 1–9 hormone-responsive elements corresponding to major phytohormones, including MeJA, ABA, SA, GA, and AUX. These results demonstrate that *SiNAC* genes are potentially regulated by complex networks of hormonal and environmental signals, highlighting their crucial roles in plant growth regulation, stress adaptation, and hormone-mediated signaling pathways.

### 2.4. Expression Patterns of SiNAC Genes Across Developmental Stages and Tissues in Foxtail Millet

**Figure 4:**
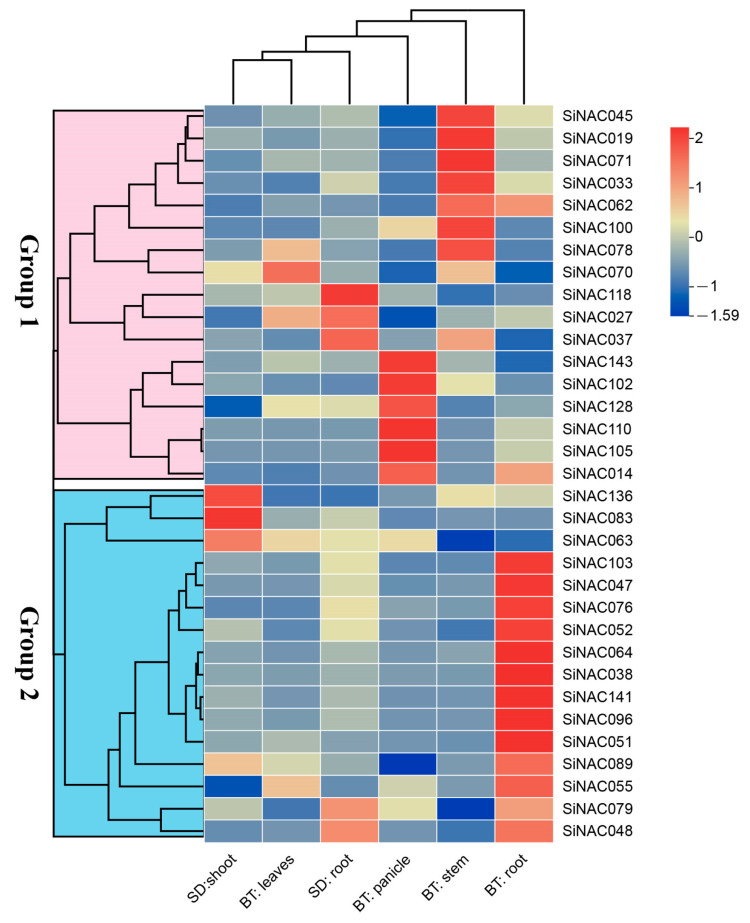
Expression profiles of *SiNAC* genes across different growth stages and tissues. Expression levels are represented by a heatmap with color-coded Z-Scores (scale shown). Developmental stages: BT, booting stage; SD, seedling stage.

A cluster heatmap was constructed to visualize the expression patterns of *SiNAC* genes across various growth stages and tissues (Figure 4). At booting stage, multiple genes exhibited preferential expression: (1) 12 genes in roots (*SiNAC103*, *SiNAC047*, *SiNAC076*, *SiNAC052*, *SiNAC064*, *SiNAC038*, *SiNAC141*, *SiNAC096*, *SiNAC051*, *SiNAC089*, *SiNAC055*, *SiNAC048*); (2) 7 genes in stems (*SiNAC045*, *SiNAC019*, *SiNAC071*, *SiNAC033*, *SiNAC062*, *SiNAC100*, *SiNAC078*); (3) 1 gene in leaves (*SiNAC070*); and (4) 6 genes in panicles (*SiNAC143*, *SiNAC102*, *SiNAC128*, *SiNAC110*, *SiNAC105*, *SiNAC014*). At seedling stage, three genes (*SiNAC118*, *SiNAC027*, *SiNAC037*) showed significantly high expression in roots, while another three genes (*SiNAC136*, *SiNAC083*, *SiNAC063*) were highly expressed in shoots.

Notably, several genes displayed high expression in two distinct tissues simultaneously. At booting stage, not only *SiNAC055* was highly expressed in leaves and roots, but also there were *SiNAC014* in panicles and roots, *SiNAC078* and *SiNAC070* in stems and leaves, and *SiNAC062* in roots and stems. Furthermore, certain genes also showed cross-stage expression patterns. For example, *SiNAC089* was highly expressed both in shoots at seedling stage and in roots at booting stage, *SiNAC037* exhibited high expression both in roots at seedling stage and in stems at booting stage, *SiNAC027* was highly expressed both in roots at seedling stage and in leaves at booting stage, and *SiNAC48* expressed consistently highly in roots across both stages.

### 2.5. Expression Profiling of SiNAC Genes During Incompatible Interaction Between Foxtail Millet and Usi 93-5

To characterize the involvement of *SiNAC* genes in disease resistance, the expression dynamics of 33 *SiNACs* in SLX leaves was evaluated at five time points (0, 12, 24, 48, and 72 hpi) following inoculation with the rust pathogen *Usi* 93-5 using qRT-PCR.

**Figure 5:**
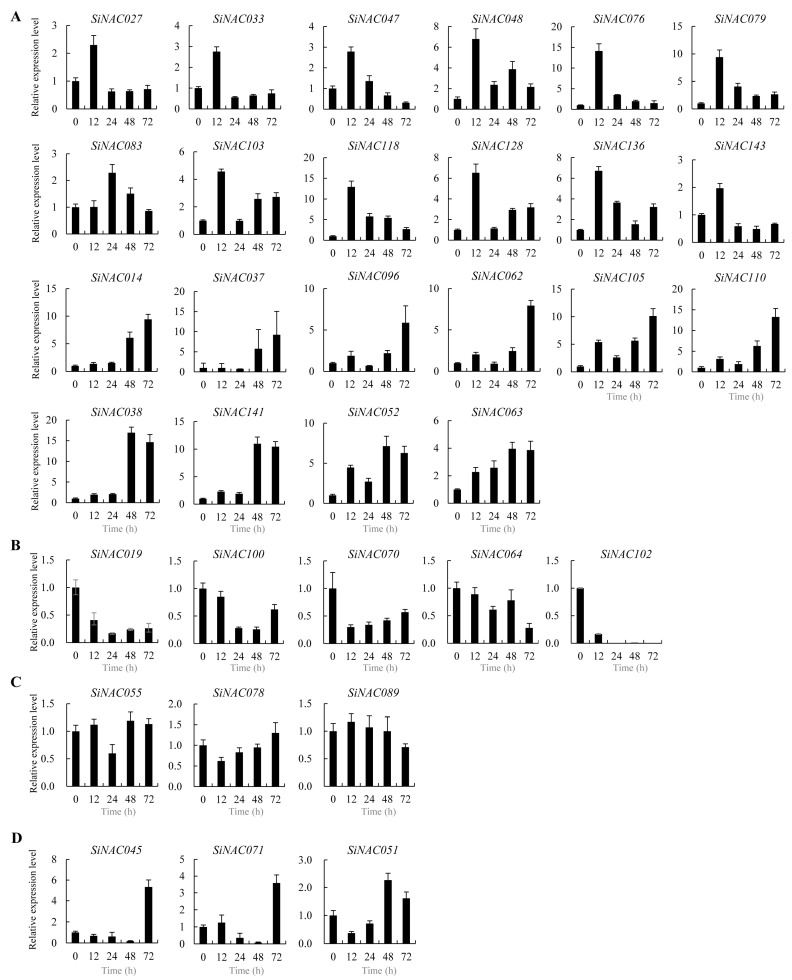
Differential expression patterns of 33 *SiNAC* genes in resistance responses. Relative expression level of *SiNACs* was calculated by the 2^−∆∆CT^ method. (**A**) *SiNAC* genes exhibiting significant upregulation. (**B**) *SiNAC* genes exhibiting significant downregulation. (**C**) Stable expression profiles of *SiNAC* genes. (**D**) Varied expression patterns of *SiNAC* genes. The vertical lines on the top of bars correspond to the standard error of the respective mean estimates.

Twelve genes (*SiNAC027*, *SiNAC033*, *SiNAC047*, *SiNAC048*, *SiNAC076*, *SiNAC079*, *SiNAC083*, *SiNAC103*, *SiNAC118*, *SiNAC128*, *SiNAC136*, and *SiNAC143*) showed significant upregulation, with most (11 genes) peaking at 12 hpi (2–14-fold increase), while *SiNAC083* exhibited delayed peak expression at 24 hpi (Figure 5A). Six genes (*SiNAC014*, *SiNAC037*, *SiNAC096*, *SiNAC062*, *SiNAC105*, and *SiNAC110*) reached maximum expression at 72 hpi, though with different induction kinetics -2 genes (*SiNAC014*, *SiNAC037*) showed activation from 48 hpi, while others (*SiNAC096*, *SiNAC062*, *SiNAC105*, *SiNAC110*) responded as early as 12 hpi. In addition, four genes (*SiNAC038*, *SiNAC141*, *SiNAC052*, *SiNAC063*) displayed peak expression at 48 hpi, maintaining stable levels through 72 hpi (Figure 5A). The analysis revealed distinct temporal regulation patterns among 22 upregulated *SiNAC* genes during the resistance response.

Five *SiNAC* genes showed significant downregulation during resistance (Figure 5B). *SiNAC102* exhibited especially dramatic downregulation (0.2 times at 12 hpi) followed by undetectable expression, suggesting potential negative regulatory functions (Figure 5B). Three genes (*SiNAC055*, *SiNAC078*, *SiNAC089*) maintained relatively stable expression at all detected time points, indicating no involvement in this resistance response (Figure 5C). Interestingly, *SiNAC045* and *SiNAC071* showed biphasic regulation with initial decline until 48 hpi followed by strong upregulation at 72 hpi, while *SiNAC051* was downregulated before 24 hpi but upregulated thereafter (Figure 5D).

These results demonstrate that different *SiNAC* members participate in distinct temporal phases of defense regulation, contributing to early (12–24 hpi), middle (48 hpi), and late-stage (72 hpi) responses during the incompatible interaction.

### 2.6. Comparative Analysis of SiNAC Gene Expression Between Incompatible and Compatible Interactions

**Figure 6:**
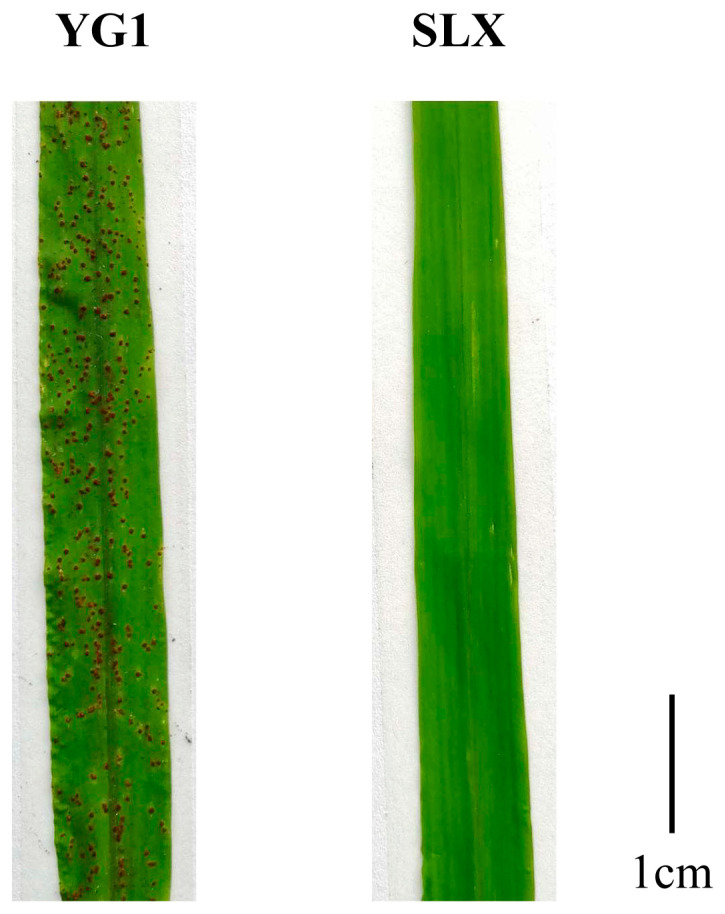
Leaf phenotypes of YG1 and SLX at 14 days post-inoculation with *Usi* 93-5. The leaves of YG1 (**Left**) and SLX (**right**) foxtail millet plants were used to inoculate *Usi* 93-5, respectively.

**Figure 7:**
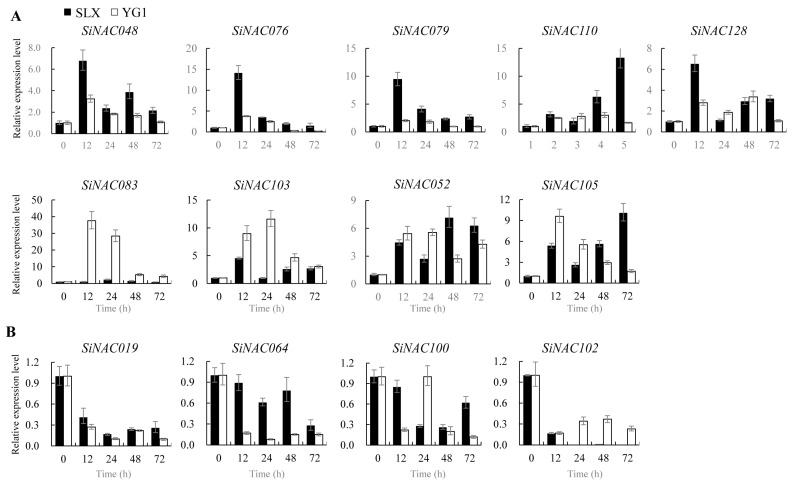
Similar expression patterns of 13 *SiNAC* genes in resistant and susceptible responses. Relative expression level of *SiNACs* was calculated by the 2^−∆∆CT^ method. (**A**) Upregulation of *SiNAC* genes in both resistance and susceptible responses. (**B**) Downregulation of *SiNAC* genes in both resistance and susceptible responses. The vertical lines on the top of bars correspond to the standard error of the respective mean estimates.

**Figure 8:**
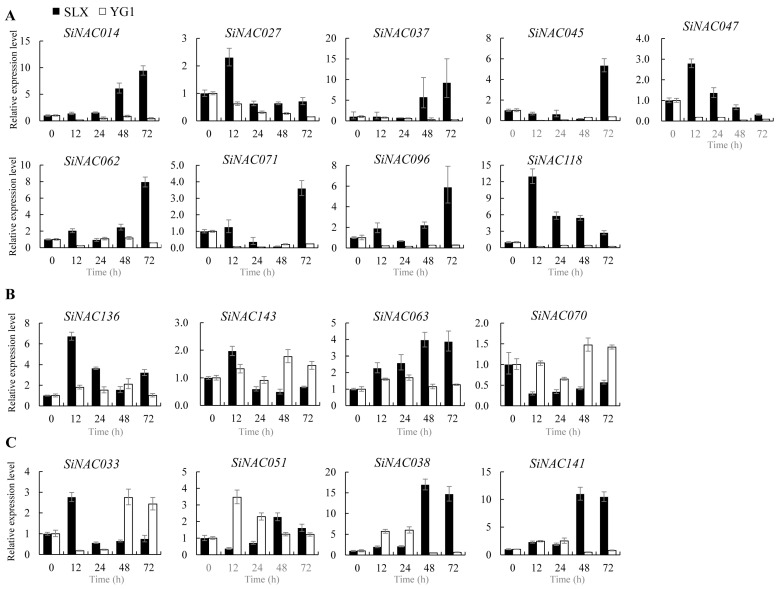
Differential expression patterns of 17 *SiNAC* genes both in resistant and susceptible responses. Relative expression level of *SiNACs* was calculated by the 2^−∆∆CT^ method. (**A**) 9 *SiNAC* genes were upregulated at some or all time points during the incompatible interaction, while their expression was downregulated in the compatible interaction. (**B**) 4 *SiNAC* genes were specifically regulated during the incompatible interaction, with changes in transcript levels ranging from induction to repression, while no notable changes occurred in the compatible interaction. (**C**) 4 *SiNAC* genes displayed distinct changes in expression dynamics at different time points in both interactions. The vertical lines on the top of bars correspond to the standard error of the respective mean estimates.

Among the 33 tested *SiNAC* genes, 30 showed altered expression during resistance, suggesting their potential involvement in defense responses. Comparative analysis of these genes’ expression between incompatible (SLX-(93-5)) and compatible (YG1-(93-5)) interactions revealed both conserved and differential expression patterns (Figure 6, Figure 7 and Figure 8).

Thirteen genes exhibited similar expression trends in both interactions (Figure 7), suggesting conserved regulatory mechanisms. Nine of these were upregulated (Figure 7A), with five showing higher expression in resistance. *SiNAC083* and *SiNAC103* displayed lower expression in resistance, while *SiNAC052* and *SiNAC105* maintained comparable levels but with different peak times—late in resistance versus early in susceptibility (Figure 7A). Four downregulated genes (Figure 7B) included *SiNAC064* and *SiNAC100* with earlier suppression in susceptibility, and *SiNAC102* showing stronger downregulation in resistance.

Seventeen genes displayed significantly different expression between resistant and susceptible interactions (Figure 8). Nine genes were upregulated during resistance but downregulated in susceptibility (Figure 8A), while four genes showed specific upregulation (*SiNAC136*, *SiNAC143*, *SiNAC063*) or downregulation (*SiNAC070*) only in resistance (Figure 8B). Four other genes exhibited complex differential patterns (Figure 8C): *SiNAC033* was strongly induced early in resistance but initially suppressed in susceptibility; *SiNAC051* showed the opposite pattern; and *SiNAC038* and *SiNAC141* were continuously induced in resistance but only transiently activated in susceptibility.

### 2.7. Functional Characterization of SiNAC Genes

Nuclear localization and transcriptional activation represent hallmark features of transcription factors. To investigate these properties in rust resistance-associated NAC proteins, we selected three candidate genes (*SiNAC063*, *SiNAC070*, and *SiNAC118*) for comprehensive functional analysis.

**Figure 9:**
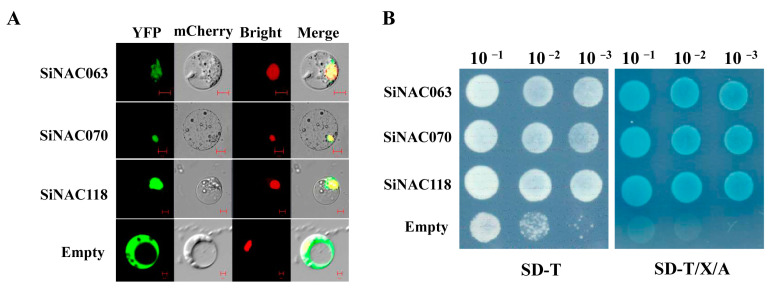
Subcellular localization and transcriptional activation analysis of *SiNAC063*, *SiNAC070*, and *SiNAC118* protein. (**A**) Fluorescence observation of *SiNAC063*, *SiNAC070*, and *SiNAC118* protein. The empty p1104-YFP vector was used as a positive control. The mCherry fused with the nuclear localization signal was used as a nuclear localization control. (**B**) Transcriptional activity of *SiNAC063*, *SiNAC070*, and *SiNAC118*. The empty pGBKT7 vector was used as a negative control.

For nuclear localization studies, we constructed C-terminal YFP fusion vectors (*SiNAC063*-p1104-YFP, *SiNAC070*-p1104-YFP, and *SiNAC118*-p1104-YFP) under the control of the CaMV35S promoter, with empty p1104-YFP vector serving as control. Following transient expression in rice protoplasts, confocal microscopy revealed distinct nuclear localization patterns for all three SiNAC-YFP fusion proteins (Figure 9A). In contrast, YFP-alone controls displayed ubiquitous fluorescence distribution throughout the cellular compartments, including nuclei, cytoplasm, and plasma membrane. These findings confirm that SiNAC063, SiNAC070, and SiNAC118 are nuclear-targeted proteins, consistent with their predicted roles as transcription factors.

Then, the full-length CDS regions of the three *SiNAC* genes were in-frame fused to the GAL4 DNA-binding domain in the pGBKT7 vector. These constructs, along with the empty pGBKT7 vector serving as a negative control, were then transformed into yeast cells. As expected, yeast colonies expressing *SiNAC063*, *SiNAC070*, and *SiNAC118* grew on selective medium (-Leu) and supplemented with Aba and X-α-gal (Figure 9B). These results indicated that *SiNAC063*, *SiNAC070* and *SiNAC118* exhibit transcriptional activation activity in yeast.

## 3. Discussion

The NAC transcription factor family represents one of the largest plant-specific transcriptional regulator groups, playing pivotal roles in plant growth, development, and stress responses [29]. Our study focused on 33 *SiNAC* genes from foxtail millet that showed differential expression under abiotic stress conditions [14]. Phylogenetic analysis classified these genes into 11 distinct NAC subgroups, revealing subgroup-specific expression patterns during rust resistance responses. Notably, genes within the NAP (*SiNAC037*, *SiNAC038*, *SiNAC108*, *SiNAC110*, *SiNAC141*), ATAF (*SiNAC048*, *SiNAC079*, *SiNAC118*, *SiNAC128*), and OsNAC3 (*SiNAC027*, *SiNAC076*) subgroups exhibited consistent upregulation, suggesting their positive regulatory roles in defense mechanisms. However, expression heterogeneity within subgroups (e.g., upregulation of *SiNAC062* and *SiNAC143*, downregulation of *SiNAC064* and *SiNAC070*, and divergent expression patterns of *SiNAC045* and *SiNAC071* within the ONAC003 subgroup) indicates that phylogenetic relationships alone cannot fully predict functional specialization.

Tissue-specific expression profiling revealed developmental stage-dependent regulation of *SiNAC* genes. During booting stage, predominant expression in roots, stems, or panicles suggests organ-specific regulatory functions, while seedling-stage expression in shoots or roots implies roles in early developmental processes. Notably, *SiNAC027*, *SiNAC055*, *SiNAC070*, and *SiNAC078* exhibit high expression levels in leaves during the booting stage. The rapid induction of *SiNAC027* at 12 hpi in the incompatible interaction, coupled with its exclusive MeJA-responsive promoter element, strongly supports its role in early defense activation. This functional conservation is evident from its rice ortholog *OsNAC4*, which confers blast resistance upon induction [30]. Conversely, *SiNAC070* showed significant downregulation during defense responses, and its promoter harbors multiple hormone-responsive elements (AUX, ABA, MeJA, GA), implicating its involvement in growth-defense coordination through hormonal crosstalk. This functional role appears evolutionarily conserved, as evidenced by its rice ortholog *OsNAC3* which regulates ABA-mediated germination and cell elongation [31]. Unlike these responsive genes, the stable expression of *SiNAC055* and *SiNAC078* during pathogen challenge, despite their promoters harboring diverse stress-responsive elements, suggests their predominant involvement in developmental regulation rather than acute defense responses. Collectively, these findings significantly advance our understanding of *SiNAC* genes’ multifaceted roles in foxtail millet development and environmental adaptation.

Previous studies on foxtail millet NAC TFs have predominantly characterized their roles in abiotic stress responses [32,33,34]. Our comparative analysis reveals complex and often contrasting expression profiles under both abiotic (drought, salinity) and biotic (*Usi* infection) stresses. For example, *SiNAC45* shows dramatic induction (25-fold) under potassium deficiency and confers stress tolerance in transgenic Arabidopsis [33], yet displays biphasic regulation during resistance response (initial downregulation followed by upregulation) while remaining suppressed in susceptible response. *SiNAC110* exhibits strong induction by dehydration (28-fold at 24 h) and salinity (70-fold at 48 h) [34], but shows differential induction during *Usi* infection (14-fold in resistant vs. 1.7-fold in susceptible responses at 72 hpi). The expression of *SiNAC78* was significantly upregulated under both drought and salt stress conditions, while remaining stable during cold stress treatment as well as during both compatible and incompatible interactions [32]. Promoter analyses reveal conserved stress-related *cis*-elements (ABA, MeJA) across these genes, with *SiNAC078* exclusively containing SA-responsive elements and *SiNAC110* uniquely harboring GA-responsive elements. This suggests distinct hormonal cascades mediate their dual roles in biotic/abiotic stress responses.

While the involvement of NAC TFs in foxtail millet–rust interactions remains unexplored, comparative analysis with wheat-stripe rust systems reveals conserved regulatory mechanisms [35]. The *TaNAC4*/*SiNAC076* ortholog pair (OsNAC3 subgroup) showed stronger induction during incompatible versus compatible interactions (wheat: 4-fold vs. 2-fold at 24 hpi; foxtail millet: 14-fold vs. 4-fold at 12 hpi), with *SiNAC076* exhibiting greater response magnitude and earlier peak expression [36]. The presence of ABA, MeJA, and GA response *cis*-elements in *SiNAC076* suggests conserved hormonal regulation. *TaNAC21/22* expression increased only during compatible interactions, and its knockdown enhanced resistance, confirming its negative regulatory role. Its foxtail millet homolog *SiNAC052* showed similar maximum induction in both interaction types but distinct temporal patterns (late-phase in resistance vs early-phase in susceptible responses), potentially mediated by tae-miR164 homologs [30].

Comparative analysis of NAC transcription factors across plant species reveals conserved molecular pathways in disease resistance. In banana (*Musa acuminata*), *MaNAC5* coordinates with *MaWRKY1/2* to activate *PR* genes during *Colletotrichum musae* infection [37]. Its foxtail millet ortholog *SiNAC063* shows resistance-specific upregulation during the incompatible interaction. Similarly, rice *OsNAC111* (TERN subgroup) enhances blast resistance through *PR* gene induction [23]. Its foxtail millet counterpart *SiNAC047* exhibits contrasting regulation (upregulated in resistant but downregulated in susceptible responses), demonstrating subgroup-specific defense functions. Promoter analyses of *SiNAC063* and *SiNAC047* identified both shared (AUX, ABA, and GA-responsive elements) and specialized *cis*-elements. Specifically, *SiNAC047* contains unique defense and stress-responsive elements, while *SiNAC063* contains a unique MeJA-responsive element. These findings indicate that *SiNAC063* and *SiNAC047* may contribute to resistance through the activation of defense-related gene (*PR*) transcription and the regulation of multiple hormone signaling pathways.

Li et al. [1] employed digital gene expression (DGE) profiling to reveal the most abundant differentially expressed signal pathways and significantly upregulated genes involved in foxtail millet’s defense response against *Usi* infection, which includes the MAPK cascades, WRKY transcription factors, NBS-LRR resistance genes, and PR proteins. Although no information is provided regarding *SiNAC* gene expression in defense response or their role in signaling pathways, we observed notable similarities in expression patterns between several *SiNAC* genes and key defense-related genes. Specifically, *SiNAC083* showed synchronized induction with WRKY transcription factor 51 (*WRKY51*) at 24 hpi. Furthermore, six *SiNACs* (*SiNAC014*, *SiNAC037*, *SiNAC038*, *SiNAC051*, *SiNAC110*, and *SiNAC141*) exhibited coordinated upregulation with *WRKY7*, *WRKY62*, *WRKY69*, *WRKY70*, and MYB transcription factor starting at 24 hpi and maintained this upregulated state thereafter. These expression correlations suggest that these SiNAC genes may participate in the regulation of key nodes within the defense signaling pathway.

## 4. Materials and Methods

### 4.1. Plant Materials, Inoculation and Sample Collection

This study employed two foxtail millet cultivars—the rust-resistant Shilixiang (SLX) harboring the *Rusi1* resistance gene [38], and the susceptible Yugu1 (YG1)—along with *Usi* pathotype 93-5. SLX demonstrated an incompatible interaction phenotype with *Usi*93-5, whereas YG1 showed complete susceptibility, establishing a compatible interaction. Fresh urediniospores of *Usi*93-5 were collected and suspended in sterile distilled water to approximately 105 cells/mL, and inoculated onto the primary leaves of 21-day-old SLX and YG1 seedings using the spray inoculation method [38]. Following inoculation, leaf tissues were systematically collected at 0, 12, 24, 48, and 72 h post-inoculation (hpi), immediately flash-frozen in liquid nitrogen, and maintained at −80 °C until RNA extraction. Three independent biological replicates were processed for each time point.

### 4.2. NAC Genes Collection and Phylogenetic Analysis

Thirty-three putative *SiNAC* genes in foxtail millet were selected for analysis based on previous literature [14], and their amino acid sequence information was obtained from the *Setaria italica* Genome Annotation (https://phytozome.jgi.doe.gov/pz/portal.html#!info?alias=Org_Sitalica, accessed on 15 May 2024) (Table S1). According to the NAC-related reports [10], we compiled predicted NAC protein sequences from *Oryza sativa* (ONAC) and *Arabidopsis thaliana* (ANAC), along with experimentally characterized NAC family members from *Oryza sativa*, *Arabidopsis thaliana*, *Petunia hybrida*, *Solanum lycopersicum*, *Nicotiana tabacum* from GenBank (https://www.ncbi.nlm.nih.gov/, accessed on 16 May 2024) and TAIR (http://www.arabidopsis.org/, accessed on 16 May 2024) (Table S2).

Predicted NAC domains of the collected SiNACs, ONACs, ANACs and known NAC family proteins were analyzed using Pfam (http://pfam.xfam.org/, release 31.0, accessed on 20 May 2024). Multiple sequence alignment was performed using the CLUSTAL W program. Phylogenetic analysis was conducted using the neighbor-joining method and bootstrap analysis (1000 replicates) [39]. The phylogenetic tree was constructed using MEGA software version 5 [40].

### 4.3. Chromosomal Distribution Analysis of the SiNACs

The chromosomal locations of all 33 *SiNAC* genes were determined using genomic coordinates from Phytozome (https://phytozome-next.jgi.doe.gov/, accessed on 26 June 2024) and NCBI’s GFF annotation file (https://www.ncbi.nlm.nih.gov/datasets/genome/GCF_000263155.2/, accessed on 27 June 2024). Their distribution patterns were visualized with TBtools [41], revealing their spatial organization across foxtail millet chromosomes.

### 4.4. Analysis of Promoter Cis-Acting Elements

The 2000 bp upstream regions of the start codons for NAC family genes in foxtail millet were extracted using TBtools. *Cis*-acting regulatory elements in these promoter regions were predicted using the PlantCARE database (https://bioinformatics.psb.ugent.be/webtools/plantcare/html/, accessed on 15 August 2024). Resultant element distributions were visualized via the Basic BioSequence Viewer module in TBtools (v2.210).

### 4.5. RNA Extraction and cDNA Synthesis

Total RNA was isolated with TRIzol Reagent (Invitrogen, Waltham, MA, USA) following the manufacturer’s protocol. To remove potential genomic DNA contamination, RNA samples were subjected to DNase I treatment. First-strand cDNA was then synthesized from DNase-treated RNA using the RevertAid^TM^ First Strand cDNA Synthesis Kit (Thermo Fisher Scientific, Waltham, MA, USA) in strict accordance with the manufacturer’s guidelines.

### 4.6. qRT-PCR Analysis

Gene expression analysis was performed by quantitative qRT-PCR (qPCR) using SYBR Premix Ex Taq II (Takara Bio, Kusatsu, Japan) on a Rotor-Gene Q thermocycler (QIAGEN, Hilden, Germany). The 20-μL reaction mixture contained 10 μL of 2× SYBR Premix Ex Taq II, 0.8 μL each of forward and reverse primers (10 μM), 2 μL cDNA template, and nuclease-free water to final volume. The thermal cycling protocol consisted of the following: initial denaturation at 95 °C for 60 s; 40 cycles of 95 °C for 10 s and 60 °C for 30 s; followed by melting curve analysis from 60 °C to 95 °C (1 °C increments) with continuous fluorescence acquisition to confirm reaction specificity. Relative transcript levels were calculated using the 2^−ΔΔCt^ method with *Act2* (*Seita.8G043100*) as the endogenous control. A ≥2-fold change in expression relative to the 0 hpi baseline was considered biologically significant.

### 4.7. Subcellular Localization of SiNACs Protein

To determine the subcellular localization of *SiNAC063*, *SiNAC070*, and *SiNAC118*, their open reading frames (ORFs) were amplified from the SLX cDNA using specific primer pairs listed in Table S3. The amplified ORFs were subsequently cloned into YFP fusion expression vectors, generating p*SiNAC063*-YFP, p*SiNAC070*-YFP, and p*SiNAC118*-YFP constructs. These constructs (p*SiNAC063*-YFP, p*SiNAC070*-YFP, and p*SiNAC118*-YFP) were co-transformed with the pSAT6-mCherry-VirD2NLS plasmid into rice protoplasts via polyethylene glycol-mediated transformation. Following a 12 h incubation in the dark at 25 °C, YFP fluorescence signals were visualized and captured using a confocal microscope (Zeiss Axio Imager.Z2, Carl Zeiss AG, Oberkochen, Germany) with an excitation wavelength of 514 nm.

### 4.8. Transactivation Assay in Yeast Cells

The transcriptional activities of three NAC genes (*SiNAC063*, *SiNAC070*, and *SiNAC118*) were evaluated in yeast cells. Their full-length coding sequences were cloned into pGBKT7 vector, respectively. An empty vector pGBKT7 was used as a negative control. All constructs were independently transformed into yeast strain Y2HGold, which harbors the AUR1-C and MEL1 reporter genes. Yeast cell transformation was performed following the protocol provided in the Yeastmaker^TM^ Yeast Transformation System 2 User Manual (Takara Bio, Kusatsu, Japan). Transformed yeast cells were cultured on SD/-Trp and SD/-Trp plates supplemented with Aba and X-α-gal plates for 2–4 days at 30 °C to evaluate transactivation activity. The primers used in this study are listed in Table S3.

## 5. Conclusions

In this study, we characterized 33 *SiNAC* genes in foxtail millet, which were phylogenetically classified into eleven distinct subgroups and unevenly distributed across nine chromosomes. Promoter *cis*-element analysis suggested their potential roles in plant growth, development, and responses to both abiotic and biotic stresses. Expression profiling revealed tissue-specific expression patterns for most *SiNAC* genes, highlighting their functional diversity. Importantly, we identified 17 *SiNAC* genes associated with rust disease resistance, which may serve as positive or negative regulators during *Usi* infection, enriching the existing disease resistance pathway. Furthermore, three of these genes were functionally validated to possess transcriptional factor characteristics. Future studies should focus on functional validation of these candidate genes and elucidation of their molecular mechanisms to enhance foxtail millet’s resistance to rust disease.

## Data Availability

All data are displayed in the manuscript and Supplementary Files.

## Supplementary Materials

The following supporting information can be downloaded at https://www.mdpi.com/article/10.3390/plants14101507/s1, Table S1. A catalog of 33 *SiNAC* transcription factor genes. Table S2. NAC proteins in *Oryza sativa*, *Arabidopsis thaliana* and other species (Ooka et al. [7]). Table S3. Primers for transactivation assay of 3 *SiNACs* in yeast.
